# Neuroplastic Effects of Combined Computerized Physical and Cognitive Training in Elderly Individuals at Risk for Dementia: An eLORETA Controlled Study on Resting States

**DOI:** 10.1155/2015/172192

**Published:** 2015-04-07

**Authors:** Charis Styliadis, Panagiotis Kartsidis, Evangelos Paraskevopoulos, Andreas A. Ioannides, Panagiotis D. Bamidis

**Affiliations:** ^1^Lab of Medical Physics, Medical School, Faculty of Health Sciences, Aristotle University of Thessaloniki, P.O. Box 376, 54124 Thessaloniki, Greece; ^2^Laboratory for Human Brain Dynamics, AAI Scientific Cultural Services Ltd., Office 501, Galaxias Building Block A, 33 Arch. Makarios III Avenue, 1065 Nicosia, Cyprus

## Abstract

The present study investigates whether a combined cognitive and physical training may induce changes in the cortical activity as measured via electroencephalogram (EEG) and whether this change may index a deceleration of pathological processes of brain aging. Seventy seniors meeting the clinical criteria of mild cognitive impairment (MCI) were equally divided into 5 groups: 3 experimental groups engaged in eight-week cognitive and/or physical training and 2 control groups: active and passive. A 5-minute long resting state EEG was measured before and after the intervention. Cortical EEG sources were modelled by exact low resolution brain electromagnetic tomography (eLORETA). Cognitive function was assessed before and after intervention using a battery of neuropsychological tests including the minimental state examination (MMSE). A significant training effect was identified only after the combined training scheme: a decrease in the post- compared to pre-training activity of precuneus/posterior cingulate cortex in delta, theta, and beta bands. This effect was correlated to improvements in cognitive capacity as evaluated by MMSE scores. Our results indicate that combined physical and cognitive training shows indices of a positive neuroplastic effect in MCI patients and that EEG may serve as a potential index of gains versus cognitive declines and neurodegeneration. This trial is registered with ClinicalTrials.gov Identifier NCT02313935.

## 1. Introduction

As humans advance through middle age and beyond brain function often changes into a mildly impaired state (mild cognitive impairment, MCI) or even deteriorates into neurodegenerative diseases like dementia and Alzheimer's disease (AD) [1, 2]. AD-related pathological changes may begin decades prior to the clinical diagnosis, with symptoms often regarded as consistent with healthy aging.

There is a growing consensus that age-related cognitive decline can be slowed down or even prevented when the brain retains its flexibility. This is underpinned by the effective reorganization of brain's structural and functional components so as to compensate for the physiological and physical changes that eventually cause cognitive decline.

Recent studies have addressed this issue in two ways: (i) developing diagnostic biomarkers that can reliably identify early signs along the continuum of MCI and AD-related pathology and if possible continuing with the longitudinal evaluation and monitoring of the progression of the disease [3–6] and (ii) modifying lifestyle behaviours to promote neuroplasticity and hence healthy aging and to prevent or at least slow down cognitive decline and AD.

Clinical MCI is among the most consistently reported risk factors for the development of AD-related pathology [7, 8] and therefore is of special interest for the aforementioned initiative. MCI patients experience greater memory loss than healthy age-matched individuals, yet they do not meet the current criteria for clinically probable AD, since they do not exhibit a resultant impairment in daily functioning [9]. Nevertheless, the annual conversion rate of MCI patients to AD is about 12%, which is significantly higher than the annual conversion rate (1%-2%) for cognitively healthy elderly individuals [10]. Not all MCI patients progress to dementia though, and some may even reverse back to healthy brain function [9, 11]. Consequently, since MCI represents a functional continuum between healthy aging and the earliest signs of dementia, it is considered suitable for possible therapeutic (nonpharmacological) interventions [9, 12].

One promising approach is to engage seniors into computerized interventions of training schemes (e.g., physical exercise and cognitive training) appropriate to their capacity [13–16]. Physical exercise serves the brain function protection and may even lead to neurogenesis across adult lifespan [17, 18]. For instance, physical exercise in older adults at risk for AD due to their diagnosis of MCI can promote stable cognitive function and increase brain volume [19]. Cognitive activity has been associated with superior cognitive functioning in healthy older adults [20] and has the potential to efficiently slow down cognitive decline in MCI patients by increasing performance on objective measures of memory [21, 22].

Changes in the functionality of the cortex at rest across the lifespan are particularly relevant to aging and neurodegeneration [23]. The resting state network (i.e., precuneus (PCu), posterior cingulate cortex (PCC), inferior parietal cortex, medial temporal lobes, medial frontal cortex, and anterior cingulate cortex [24–26], also called default mode network (DMN) [27]) is generally vulnerable to atrophy [28]. The disruption of DMN's functionality is correlated with amnestic MCI (aMCI) [29] and AD [30] and is thus related to the severity and the progression of neurodegeneration [31].

One of the major effects along the continuum of MCI and AD conditions is electroencephalogram (EEG) “slowing” [32–34]. Resting state EEG rhythms in MCI/AD show an increase of power in low frequencies (delta and theta band, 0.5–8 Hz) and a decrease of power in higher frequencies (alpha and beta, 8–30 Hz), supporting the transition theory from healthy aging to AD with MCI being an intermediate state [35, 36]. Moreover, the posterior sources of delta and dominant alpha rhythms are related to global cognitive status (i.e., MMSE score) in both MCI and AD subjects [37].

Though the cortical sources of resting state eyes-closed EEG rhythms can be sensitive markers for tracking the progression of MCI's underlying neurodegenerative processes, these markers have not been used so far to investigate the beneficiary changes of a combined intervention scheme of physical and cognitive training in MCI patients. We hypothesized that such a training scheme can potentially index the slowing down of the typical alterations of MCI in the EEG rhythms; these resting state changes are expected to be superior for the combined intervention due to its possibly additive benefits [38–41]. We tested this hypothesis in a resting state study of 70 seniors where resting state eyes-closed EEG data were recorded before and after an eight-week intervention of cognitive and/or physical training. In order to confirm the effect of the combined training we repeated the analysis on an additional sample of 14 MCI patients.

## 2. Materials and Methods

### 2.1. Participants

**Figure 1:**
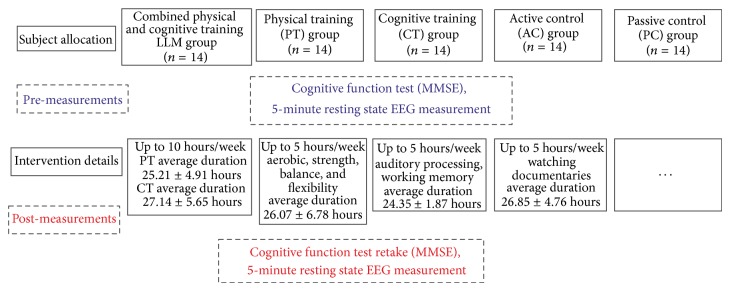
Flow of participants within the 3 experimental and 2 control groups.

This is a longitudinal study involving 70 (25 male) right handed MCI individuals (mean age = 70.80; SD = 5.67) (Figure 1). All of the participants went through a neuropsychological assessment which was part of the screening process for the Long Lasting Memories (LLM) project (http://www.longlastingmemories.eu/). Screening took place 1–14 days before the participants' enrolment to the training [42]. They were divided into 5 equally populated groups (14 participants per group) that underwent interventions following the distinct training types of the LLM project. The protocol was approved by the Bioethics Committee of the Medical School of the Aristotle University of Thessaloniki, as well as the Board of the Greek Association of Alzheimer's Disease and Related Disorders (GAADRD). Participants provided written informed consent prior to study participation. Prior to neurophysiological acquisition, the participants were informed that they could terminate the experiment at any time without the need to provide any justification for their decision (no one did). LLM's interventions took place at the GAADRD clinic. The LLM project was conducted in accordance with the Helsinki Declaration for Human Rights.

#### 2.1.1. Neuropsychological Examination

The neuropsychological examination consisted of tests allowing assessment on the participant's generic cognitive status and other specific cognitive domains (verbal memory, executive functions, independent living, etc.) that are essential to the diagnostic procedure and the group formation. Further details are available in Bamidis [43].

#### 2.1.2. Diagnostic Procedure

A dementia expert neurologist, naïve regarding the treatment each subject received, performed the diagnosis of the participants taking into consideration the neurophysiological as well as the medical examination [44]. MCI patients met Petersen's criteria based on subjective and objective cognitive impairment, predominantly affecting memory, in the absence of dementia or significant functional loss [9, 12]. All MCI participants had a Clinical Dementia Rating score of 0.5 [45].

#### 2.1.3. Cognitive Status Test

The minimental state examination (MMSE) is a brief, 30-point measure that is used to assess cognitive status [46]. MMSE is routinely used in clinical practice to screen for dementia, but here it only served as an index of patient's response to treatment. Specifically, it is used to estimate the severity of cognitive impairment and to follow the longitudinal cognitive changes in a patient. Normal cognition MMSE score is greater than or equal to 27 points. Various proposals on the score level to be used as the cut-off point for the diagnosis of dementia have been made (i.e., 23/24 [47] or even 20/21 [48]). In spite of the methodological differences, the cut-off value of 23/24 [49] has been regarded as a valid cut-off level for the diagnosis of dementia in Greece [50].

#### 2.1.4. Inclusion Criteria for the Current Study

They are as follows: (i) ages ≥ 60 years, (ii) 23 ≤ MMSE score ≤ 27 points, (iii) normal or corrected-to-normal hearing and vision, (iv) fluent language skills, and (v) agreement of a medical doctor and time commitment to the intervention protocol.

#### 2.1.5. Exclusion Criteria

They are as follows: (i) unrecovered neurological disorders (i.e., stroke, traumatic brain injury), (ii) severe depression or psychological disorder, (iii) unstable medication within the last 3 months, (iv) severe physical disorder, and (v) concurrent participation in another study.

#### 2.1.6. Matching

The participants of each group were matched on age, years of education (yoe), and male-to-female ratio, as well as cognitive state as screened by the MMSE [46] (see Table 1).

#### 2.1.7. Categorization

Participants of the LLM group (mean age = 71.21; SD = 4.52; mean MMSE = 25.85; SD = 2.09; mean yoe = 8.14; SD = 3.06; 5 males) attended a training protocol consisting of physical and cognitive exercises. Participants of the physical training (PT) group underwent only physical training (mean age = 70.42; SD = 6.63; mean MMSE = 26.21; SD = 2.33; mean yoe = 6.14; SD = 1.45; 5 males) whereas participants in the cognitive training (CT) group performed cognitive tasks (mean age = 72.71; SD = 6.57; mean MMSE = 25.14; SD = 3.22; mean yoe = 6.14; SD = 3.22; 5 males). Moreover, two control groups were employed: the active control group (AC) (mean age = 71.07; SD = 4.38; mean MMSE = 26.21; SD = 1.97; mean yoe = 7.14; SD = 3.04; 5 males) in which participants underwent a training protocol consisting of watching a documentary and answering a questionnaire and a passive control group (PC) (mean age = 67.64; SD = 3.97; mean MMSE = 25; SD = 1.77; mean yoe = 7.35; SD = 2.37; 5 males) in which participants did not engage in any activity.

### 2.2. Long Lasting Memories (LLM) Intervention

LLM is an integrated training system that targets nondemented and demented aging population and adopts an approach of cognitive [51] and physical training [52, 53] in order to improve the quality of life and prolong the functionality of the elders. All training components of the intervention were computerized, centre-based, and under supervision. The combined cognitive and physical training sessions were performed in a pseudorandomized counterbalanced sequence. The details of each training intervention are described in detail in [43, 51, 53, 54] and are summarized in Figure 1 and Table 1. Our experimental design allows for the exploration of the distinct mechanisms crucial for transferring the combined training effects. The trial was registered retrospectively (ClinicalTrials.gov Identifier: NCT02313935). This was a result of strict project timeline but also unclear areas of responsibility in the project (as the trial did not involve any medicinal products covered by Directive 2001/20/EC, guidelines from the European Medicines Agency and Eudra CT specifically indicated that there was no legal obligation from the sponsor to register it into a trial database).

#### 2.2.1. Cognitive Training (CT)

The CT component of LLM is a Greek adaptation of the Brain Fitness software (Posit Science Corporation, San Francisco, CA, USA). It employs auditory stimuli and comprises six exercises of self-paced levels of difficulty. Each exercise lasted fifteen minutes. Each CT session consisted of four out of six exercises with an overall duration of one hour. CT was performed for one hour per day, three to five days per week during a period of eight weeks. CT targeted auditory processing and working memory [55]. Details on the benefits of auditory training on age-related cognitive decline are discussed elsewhere [56].

#### 2.2.2. Physical Training (PT)

The PT component of LLM, FitForAll (FFA) [53], is an elderly tailored environment [52] where physical exercise is blended by games (exergaming) with the use of supporting hardware like Nintendo Wii, Wii remote, and Wii balance-board, to enable an enjoyable digital training experience. PT was performed for five sessions per week one hour per day during a period of eight weeks. PT was performed in the context of computer-based games which were appropriately adjusted to elder's capacity. The games' scenarios targeted body flexibility, balance, and strength as well as physical endurance through aerobic training. Each participant had to accomplish 20 minutes of aerobic exercises, 8–10 resistance exercises, 10 minutes of flexibility exercises, and a set of balance targeted exercises. The warm-up and cool-down processes constituted the initial and final session's components, respectively. The effects of combined aerobic and strength exercise which is thought to be the most effective exercise training for improving cognitive function are discussed elsewhere [15, 57, 58].

#### 2.2.3. Active Control (AC)

AC aids in controlling for potential confound factors such as willingness to adopt an active aging profile, computer skills, and social interaction [51]. In the current study, though the participants in AC group were exposed to similar training parameters (e.g., computer use, intensity, and duration), they just viewed documentaries on nature, art, and history and completed questionnaires about the documentaries [59]. AC did not involve any PT.

### 2.3. Experimental Design and EEG Recording 

The pre- and post-intervention EEG recordings were performed under medical supervision using a Nihon Kohden EEG device with 57 scalp electrodes and a sampling rate of 500 Hz. Electrode impedances of brain signals, ground electrode, and references were kept lower than 2 kΩ. Five-minute resting state (eyes closed) EEG signals were recorded prior to the initiation of the intervention phase (8 weeks) and following its completion. Participants were instructed to keep their eyes closed and to maintain a resting yet wakeful condition. Active scalp electrodes were placed on a cap (EASYCAP, http://www.easycap.de/easycap/) according to the 10–20 system. The electrodes were commonly referenced to the average of the two linked mastoid electrodes. EOG signals were recorded simultaneously by means of four Ag/AgCl electrodes (one above and one below the right eye and another two placed at the outer canthi of each eye). The vertical EOG (VEOG) was calculated as the difference between the two signals recorded above and below the right eye, while the horizontal EOG (HEOG) was calculated as the difference between the signals recorded from the left and the right electrodes, respectively. ECG signal was also recorded simultaneously by means of two Ag/AgCl electrodes.

### 2.4. Data Preprocessing and Resting State Neuroimaging

The data preprocessing was performed via the FieldTrip toolbox for MATLAB [60]. The EEG recordings were filtered using a high-pass IIR filter at 1 Hz, a notch IIR filter at 48–52 Hz, and a low-pass IIR filter at 97 Hz. Independent component analysis (ICA) was applied to the filtered EEG data for removing EOG and ECG artefacts [61]. The ICA components were visually inspected and the artifactual ones were removed. The EEG recordings were further inspected after the removal of the ICA components and all remaining visible artefacts were removed. A random process was used to select 15 segments each one with duration of 4 seconds from each EEG recording. The selected segments were imported to sLORETA/eLORETA software [62]. The 15 segments were transformed to the frequency domain in cross-spectral form. The exact low resolution brain electromagnetic tomography (eLORETA) [62] current density reconstructions (CDR) were calculated and projected on a generic MNI-152 head model separately for delta (2–4 Hz), theta (4–8 Hz), alpha (8–12 Hz), beta 1 (12–18 Hz), and beta 2 bands (18–30 Hz). Finally, the resulting eLORETA images of each participant were normalized by scaling the total average power equal to unity [63].

### 2.5. Statistics

The statistical nonparametric mapping (SnPM) method as implemented in sLORETA/eLORETA software package was used to perform the statistical analyses [64]. The empirical probability distribution of the maximum *F* statistic was estimated via randomization, under the null hypothesis of equality between pre- and post-intervention, for each discrete frequency band within the groups. The same analysis was performed on an additional sample (*n* = 14) of MCI patients who received the combined training so as to check whether our results would be replicated. For the between-groups comparison, we estimated, via randomization, the empirical probability distribution of the maximum logarithm of the *t* statistic under the null hypothesis of equality of the difference between pre- and post-measurements of one group to another, for each discrete frequency band. This methodology corrects for multiple testing for all discrete frequencies [65]. Due to the nonparametric nature of the method, its validity need does not rely on any assumption of Gaussianity [64]. For the comparisons to the mean cortical activations, we used SnPM8b (http://warwick.ac.uk/snpm). Nonparametric Spearman's rho was used to test the correlation between the MMSE pre- to post-difference of each participant and the activity of a region of interest (ROI) that included only the current density cluster showing significant differences before to after training.

## 3. Results

### 3.1. Neurophysiological Measurements

**Figure 2:**
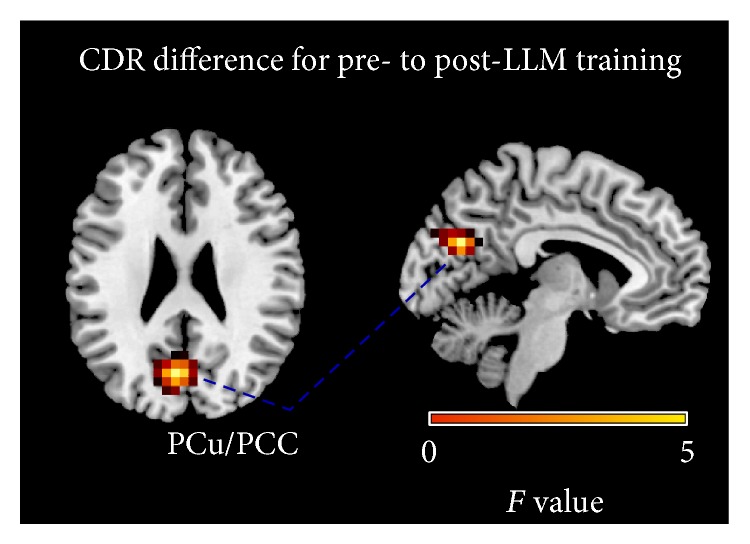
Grand average of eLORETA solutions (i.e., CDR at PCu/PCC voxels at *P* < 0.05, corrected) modelling the EEG source for delta band in the LLM group on the corresponding axial (left view) and sagittal (right view) generic MRI slices. The left side of the maps (left view) corresponds to the left hemisphere. The power estimate was scaled based on the averaged maximum value indicated in the scale bar. Similar illustrations but of fewer voxels apply for the theta, beta 1, and beta 2 bands.

Statistical analyses on the individual differences of eLORETA images were performed, comparing the source CDRs before and after the intervention within each group. The analyses revealed that only the LLM group had statistically significant differences on several frequency bands. Specifically, we observed a decrease of cortical activity for delta (peak coordinates: *x* = 0, *y* = −75, and *z* = 20; *P* < 0.05 corrected), theta (peak coordinates: *x* = 0, *y* = −75, and *z* = 20; *P* < 0.05 corrected), beta 1 (peak coordinates: *x* = −5, *y* = −75, and *z* = 20; *P* < 0.05 corrected), and beta 2 bands (peak coordinates: *x* = −5, *y* = −75, and *z* = 15; *P* < 0.05 corrected), all localized in the PCu extending in the PCC. We did not find significant differences in the alpha band. For illustrative purposes, Figure 2 maps the grand average of the eLORETA solutions (i.e., relative current density at PCu/PCC) for the LLM group modelling the EEG source only for the delta rhythm. The results of the additional sample of the LLM group are very similar to our main results (see Supplementary Material available online at http://dx.doi.org/10.1155/2015/172192). The differences between groups were compared in order for the effect of each training element to be determined. Specifically, we compared the pre- and post-training source CDRs of the LLM group to the pre- and post-CDRs of the CT and the PT group. Our results revealed a statistically significant difference of cortical activity on theta band for the comparison of the LLM to the PT group at the PCC (peak coordinates: *x* = −5, *y* = −70, and *z* = 10; *P* < 0.05 corrected). The results are described in detail in Table 2.

### 3.2. MMSE Measurements

**Figure 3:**
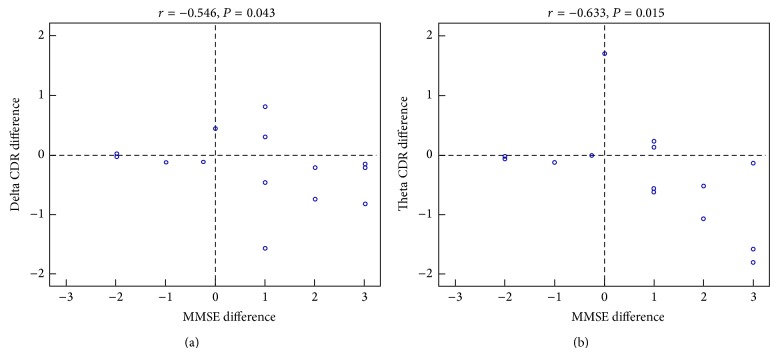
Visualization of the negative correlation of delta (*r* = −0.546, *P* = 0.043) (a) and theta (*r* = −0.633, *P* = 0.015) (b) bands among the MMSE score post- to pre-difference of each participant and the CDR post- to pre-difference of PCu/PCC activity that was statistically significant at *P* < 0.05, corrected.

We performed a paired *t*-test on the pre- to post-MMSE scores for each group and though the mean direction of change for all groups was an increase (see Table 1), none of the comparisons reached significance. Moreover, we performed a correlation analysis between the MMSE score of each participant and the neurophysiological activity of a ROI that included only the cluster showing significant differences post- to pre-training. This analysis indicated that the source current density difference of post- to pre-training of the LLM group had a significantly negative correlation to MMSE score difference in delta (*r* = −0.546, *P* = 0.043) and theta bands (*r* = −0.633, *P* = 0.015) (Figure 3): the greater the decrease of activity due to the training in this ROI for both delta and theta, the higher the improvement in the MMSE. Similar results were obtained for the additional sample of the LLM group (see Supplementary Material).

## 4. Discussion

We demonstrate that, after an eight-week long intervention of combined physical activity and cognitive training in MCI patients, a resting state change of brain activity as measured via EEG emerges showing that (i) combined training significantly decreases delta, theta, and beta rhythms, (ii) PCu/PCC activity decrease implies functional plasticity, (iii) the greater the delta and theta decrease of activity, the higher the improvement in the MMSE, (iv) short-term interventions of both physical and cognitive trainings can significantly tap into brain plasticity, and (v) physical activity may play a crucial role in transferring the combined training effects.

### 4.1. Combined Training in MCI Significantly Decreases Delta, Theta, and Beta Rhythms

Increases in delta and theta power are consistent changes in the continuum along MCI and AD conditions [66, 67]. With reference to healthy elderly subjects, MCI patients exhibit theta increases [68, 69], whereas delta increases are more evident in AD patients [36, 70–72]. Longitudinally, theta power is found to be particularly high in baseline evaluations of MCI patients who show decline at a 7-year follow-up [73]. We interpret the delta and theta decreases in MCI patients, induced by the combined training, as a beneficiary neuroplastic outcome that may index a possible deceleration of the underlying neurodegeneration. Our results show promise given that delta occipital sources present a progressive decreasing trend with physiological aging [74] and age is inversely related to the amount of slow activity (delta/theta), indicating that increase of slow activity is not a marker of physiological aging [75]. Moreover, as theta band is highly correlated with loss of memory function in MCI, its increase may index memory capacity deterioration [69, 76]. In addition, delta and theta increases are strictly related to the bilateral reduction of memory circuits (e.g., hippocampus, entorhinal volumes) of AD patients [77, 78] and global gray matter volume is inversely related to the power of pathological delta sources in MCI and AD [79].

Our beta band findings are not consistent with evidence that MCI progression to dementia is usually characterized by beta band decrease [35, 36]. We could speculate that either the tomographic and analysis methods used herein could not reveal such an effect or beta band is not yet impaired in our sample (its impairment could be expressed later). Nevertheless, we found beta 1 and beta 2 decrease only in the PCu/PCC. Given the wide variations in beta activity within groups (e.g., beta 2 is highest in the healthy controls and decreases in the aMCI and AD patients in the frontal and temporal scalp regions, whereas beta can vary among these groups [80]), it appears that beta band population properties are less consistent among the MCI pathology and possibly specific to certain lobes [81]. Overall, our results for delta, theta, and beta bands are in line with reports that MCI patients with increased MMSE scores have relatively less amplitude in delta, theta, and beta 1 rhythms than those who have decreased their MMSE scores [81]. The exciting implication of our results is that MCI patients may decelerate the progress of dementia with combined short-term (but intense) physical and cognitive training.

### 4.2. Decrease of PCu/PCC Activity Implies Reorganization of the Surviving Neuronal Circuitries

The beneficiary changes observed on EEG rhythms in the MCI patients of the LLM group were localized in the PCu/PCC (BA 31), which is positioned between the cingulate and splenial sulci and belongs to both PCC and PCu cortices [82, 83]. PCu/PCC is a main hub of the DMN [84] and shares structural connections with many areas (prefrontal, premotor, and supplementary motor areas [84], inferior parietal cortex [85], medial temporal lobe [86], and hippocampus [87, 88]). This structural connectivity is disrupted in MCI [88] and AD [87].

PCu and PCC hypometabolism as well as that of temporal and parietal regions is among the recently proposed markers for AD diagnosis [5, 89–92]. It accompanies neurodegeneration along the continuum of MCI and AD, a pattern that is often distinct from normal aging [93–97]. Also, single-photon emission computed tomography (SPECT) studies show that hypoperfusion in parietal and temporal lobe regions and in the PCu may be brain functional patterns occurring very early in AD [98, 99].

Delta/theta rhythms and perfusion/metabolism are inversely correlated in temporoparietal regions of AD patients [98–100]. An increase in theta power associates with cerebral ischemia [101], decreased glucose metabolism in temporoparietal regions [102], and decreased hippocampal volume [103]. Decreased glucose metabolism is linked to increases in delta power band as well [104]. Brain regions demonstrating excess slow-wave activity are underperfused [105]. For instance, MCI patients at lower risk to develop AD, who have a constant trend toward a higher brain regional blood perfusion, maintain low levels of hippocampal theta power [106]. Furthermore, abnormalities in slow EEG rhythms and alterations in perfusion/metabolism correlate with severity of AD as expressed by MMSE [71, 107, 108]. Given the relationship between functional neuroimaging (functional magnetic resonance imaging (fMRI), PET, and SPECT) and scalp-recorded EEG, our findings suggest that the spatial characteristics of the EEG rhythms may contain relevant information regarding the slowing down of neurodegeneration in MCI.

### 4.3. Delta and Theta Rhythms Decrease Is Correlated with MMSE Score Increases

The inverse correlation between posterior delta power and the MMSE score of healthy elders as well as MCI and AD patients indicates that improvement of the global cognitive status is related to a decrease of pathological delta rhythms [37]. A similar correlation exists for the worsening of cognitive functions over AD progression [34]. These findings agree with the bulk of previous evidence on the enhancement of the delta rhythms in MCI and AD patients compared to healthy elders [35, 37, 66]. In the current study, our results on the cognitive status of MCI patients of the LLM group imply that the greater the delta and theta decrease of activity due to the training in the PCu/PCC, the higher the improvement in the MMSE. No other band in none of the other groups exhibited a correlation with the MMSE in our study. Thus, though it seems that MMSE can significantly improve over time even due to short-term changes in lifestyle involving cognitive and physical training, the extraction of concrete conclusions regarding the sustainability of the intervention effects on the cognitive capacity of the participants can be reached only via follow-up measurements.

### 4.4. Short-Term Interventions of Both Physical and Cognitive Training Can Significantly Tap into Brain Plasticity

The EEG decrease in PCu/PCC observed here is a beneficiary outcome of the short-term, intensive, and combined intervention in MCI patients as it implies functional reorganization and plasticity in an area that is often used as one of the first indices of neurodegeneration [30, 109]. This area is important for internal processing [84] which means that its observed improvement may result in an efficient translation of internally represented goals that relate to physical and/or cognitive stimulation to actions. From this perspective, our combined intervention scheme has the potential to successfully modify lifestyle behaviours of our pathological aging population.

As a main hub of DMN, PCu/PCC may constitute common ground for both physical and cognitive training effects in older populations. Recent functional studies indicate an increase of the resting state BOLD signal of this area after cognitive training in the elderlies [110, 111]. Similarly, a higher regional cerebral blood flow in the PCu/PCC of master elderly athletes was found as a result of life-long aerobic exercise in comparison to sedentary older adults [106] and this was interpreted as being a manifestation of preserved blood supply targeting the PCu/PCC against age-related degradation. Also, physical activity was positively correlated with PCu volume in healthy elders [107, 108].

Our findings support that combined interventions, occurring either sequentially or simultaneously, show promise in maintaining or improving cognitive functions [13, 112–114]. Combined training can improve general cognitive performance and subjective measures of functional status as compared to a no-treatment control and is more promising than the single training groups [41]. Advantages conferred by combined interventions emerge from the beneficial effect of physical activity on brain metabolism, but this metabolic benefit can be put to use only if a cognitive effort (e.g., cognitive training) is performed [41]. The other experimental (CT, PT) and control groups (AC) did not show significant alterations in their cortical activity after training. Our interpretation of this finding is that both the active-control activities and the placebocontrol activities may be beneficial to the participants and may have potential impact on the outcomes, but nevertheless, their impact is not adequate to reach significance.

### 4.5. Mild Physical Activity Drives the Improvement in the Combined Training

The significant differences between the LLM and the PT group reveal that the mild physical activity (aerobic, resistance, body flexibility, and balance training) plays a crucial role in transferring the combined training effects even when occurring in a short-term period. Our finding supports that aerobic training is the core mechanism in cognitive ability enhancement [115]. In contrast, it contradicts that aerobic training must be practiced for at least one consecutive year to produce cognitive benefits in elders [116]. Thus, though the duration of the physical activity is short in our intervention, it is plausible that its nature of both aerobic and resistance training and body flexibility and balance are important factors for the manifestation of the neuroplasticity changes. Indeed, combined aerobic and strength training has a greater effect compared to single mode exercise training in cognitively nonimpaired [15] and cognitively impaired older adults [117].

### 4.6. Strengths and Limitations

The main strengths of the present study are the 5-group design and a well-distributed social support across all intervention groups. One limitation of the study is the fact that it was not conducted in a blinded fashion. Moreover, the small sample size (*n* = 14) per group might have limited the intervention effects. Nevertheless, since the results of the additional sample of the LLM group are quite similar to our main results (see Supplementary Material), we are confident for the validity and replicability of our promising results. Finally, the lack of clinical follow-up of our MCI patients does not provide further interesting insights on whether the combined training is an adequate measure to decelerate or prevent conversion to AD. We note, despite the limitations, that our findings support the claim that physical exercise and cognitive stimulation have the potential to improve cognitive performance in cognitively pathological populations.

## 5. Conclusion

Our study brings forth new insights on the benefits from combined physical exercise and cognitive training by revealing beneficiary neuroplasticity changes across elderly individuals at risk for AD. Here, we provide evidence of changes that are realized after only eight weeks of intensive training. The implication is that even short training combining cognitive and physical components has the potential to significantly improve daily life functioning. Our findings suggest that EEG can be considered as a potential index of slowing MCI through a nonpharmaceutical intervention as the one employed here. Nevertheless, we acknowledge that the resulting brain patterns are primarily correlational and, therefore, more studies are needed to elucidate how the combined intervention induced the improvement [13, 118].

## Supplementary Material

The supplementary material contains the results of the exact same to the main paper analysis for the additional sample of 14 MCI patients who received the combined training. It also includes the figures (Supplementary Figure 1, and 2) and tables (Supplementary Table 1, and 2) related to these results.

## Figures and Tables

**Table 1:** Subject pool (means ± SDs) and training type details.

	LLM	PT	CT	AC	PC
Number of subjects	14	14	14	14	14
Number of males/ratio	5 (35.71%)	5 (35.71%)	5 (35.71%)	5 (35.71%)	5 (35.71%)
Age	71.21 ± 4.52	70.42 ± 6.63	72.71 ± 6.57	71.07 ± 4.38	67.64 ± 3.97
Pre-MMSE	25.85 ± 2.09	26.21 ± 2.33	25.14 ± 3.22	26.21 ± 1.97	25 ± 1.77
Post-MMSE	27.14 ± 2.06	27.42 ± 2.06	25.42 ± 2.35	27.28 ± 1.97	25.21 ± 2.42
yoe	8.14 ± 3.06	6.14 ± 1.45	6.14 ± 3.22	7.14 ± 3.04	7.35 ± 2.37

Intervention details	PT and CT	Aerobics, strength, balance, and flexibility	Auditory processing and working memory	Watching documentaries on YouTube	—
Sessions	Up to 10 h/w	Up to 5 h/w	4 exer × 15 min, 3 to 5 h/w	Up to 5 h/w	—
Duration	PT: 25.21 ± 4.91 h CT: 27.14 ± 5.65 h	26.07 ± 6.78 h	24.35 ± 1.87 h	26.85 ± 4.76 h	—

Note: LLM, combined training; PT, physical training; CT, cognitive training; AC, active control; PC, passive control; MMSE, minimental state examination (where the range from best to worst performance is 30–0); yoe, years of education; exer, exercise; min, minutes; h, hour; w, week.

**Table 2:** EEG source maps at the differences at *P* < 0.05, corrected.

LLM					
Band	Anatomical area	BA	CS	*F*	MNI coordinates (mm) *x*, *y*, *z*
Delta (2–4 Hz)	PCu/PCC	31	56	3.9294	0, −75, 20
Theta (4–8 Hz)	PCu/PCC	31	9	2.7361	0, −75, 20
Beta 1 (12–18 Hz)	PCu/PCC	31	10	2.9856	−5, −75, 20
Beta 2 (18–30 Hz)	PCu/PCC	31	15	3.3563	−5, −75, 15

LLM versus PT					
Band	Anatomical area	BA	CS	*T*	MNI coordinates (mm) *x*, *y*, *z*

Theta (4–8 Hz)	PCC	30	1	4.1581	−5, −70, 10

Note: results are superimposed on standardized MNI coordinates; BA, Brodmann area; *x*, left/right; *y*, anterior/posterior; *z*, superior/inferior; CS, cluster size in number of activated voxels; *F*, *F* value; *T*, *t* value; PCu/PCC, precuneus/posterior cingulate cortex; significant at *P* < 0.05, corrected.
