# Supplementary material for: Neuroplastic Effects of Combined Computerized Physical and Cognitive Training in Elderly Individuals at Risk for Dementia: An eLORETA Controlled Study on Resting States
Source: Neural Plast. 2015 Apr 7;2015:172192. doi: 10.1155/2015/172192 (PMC4405298; doi:10.1155/2015/172192)
Supplement: Supplementary file 1 — The supplementary material contains the results of the exact same to the main paper analysis for the additional sample of 14 MCI patients who received the combined training. It also includes the figures (Supplementary Figure 1, and 2) and tables (Supplementary Table 1, and 2) related to these results. [file 172192.f1.docx]

**Results of the additional LLM sample**

In order to confirm the effect of the combined training we repeated the analysis on an additional sample of 14 MCI patients (see Supplementary Table 1). The decrease of cortical activity for delta (peak coordinates: x=25, y=-75, z=20; p<0.05 corrected), theta (peak coordinates: x=25, y=-75, z=20; p<0.05 corrected), and beta1 bands (peak coordinates: x=20, y=-75, z=20; p<0.05 corrected), was localized in PCu/PCC. In addition, the increase of beta2 band (peak coordinates: x=45, y=-10, z=-5; p<0.05 corrected) was localized in the superior temporal gyrus. This increase can also be regarded as a beneficiary neuroplastic outcome given that MCI progression to dementia is usually characterized by beta band decrease [1,2], for instance in the temporal and occipital lobes [1]. These results are described in detail in Supplementary Table 2.We did not find significant differences in the alpha band. For illustrative purposes, Supplementary Figure 1 maps the grand average of the eLORETA solutions for the LLM group modelling PCu/PCC only for the theta rhythm. Finally, we found that the source current density difference of post to pre training for the additional sample of the LLM group had a significantly negative correlation to MMSE score difference in delta (r = -0.681, p = 0.007) and theta bands (r = -0.553, p = 0.04) (Supplementary Figure 2).

References

[1] V. Jelic, S. E. Johansson, O. Almkvist, M. Shigeta, P. Julin, A. Nordberg, B. Winblad, and L. O. Wahlund, “Quantitative electroencephalography in mild cognitive impairment: longitudinal changes and possible prediction of Alzheimer’s disease,” *Neurobiol. Aging* **21**, 533–540 (2000).

[2] C. Huang, L. O. Wahlund, T. Dierks, P. Julin, B. Winblad, and V. Jelic, “Discrimination of Alzheimer’s disease and mild cognitive impairment by equivalent EEG sources: a cross-sectional and longitudinal study,” *Clin. Neurophysiol.* **111**, 1961–1967 (2000).

**Supplementary Figures**

**Supplementary Figure 1.**

**
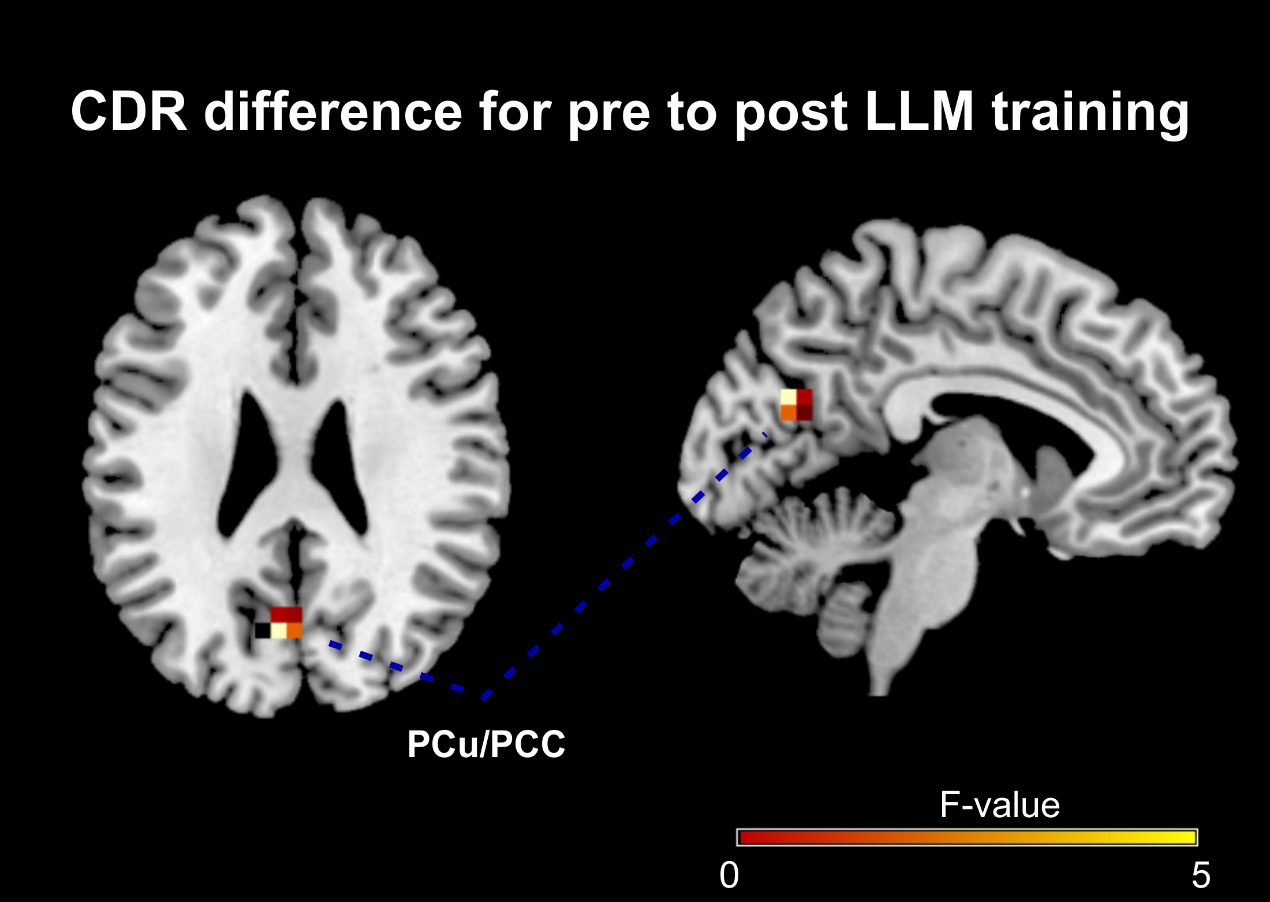
**

**Supplementary Figure 2.**

**
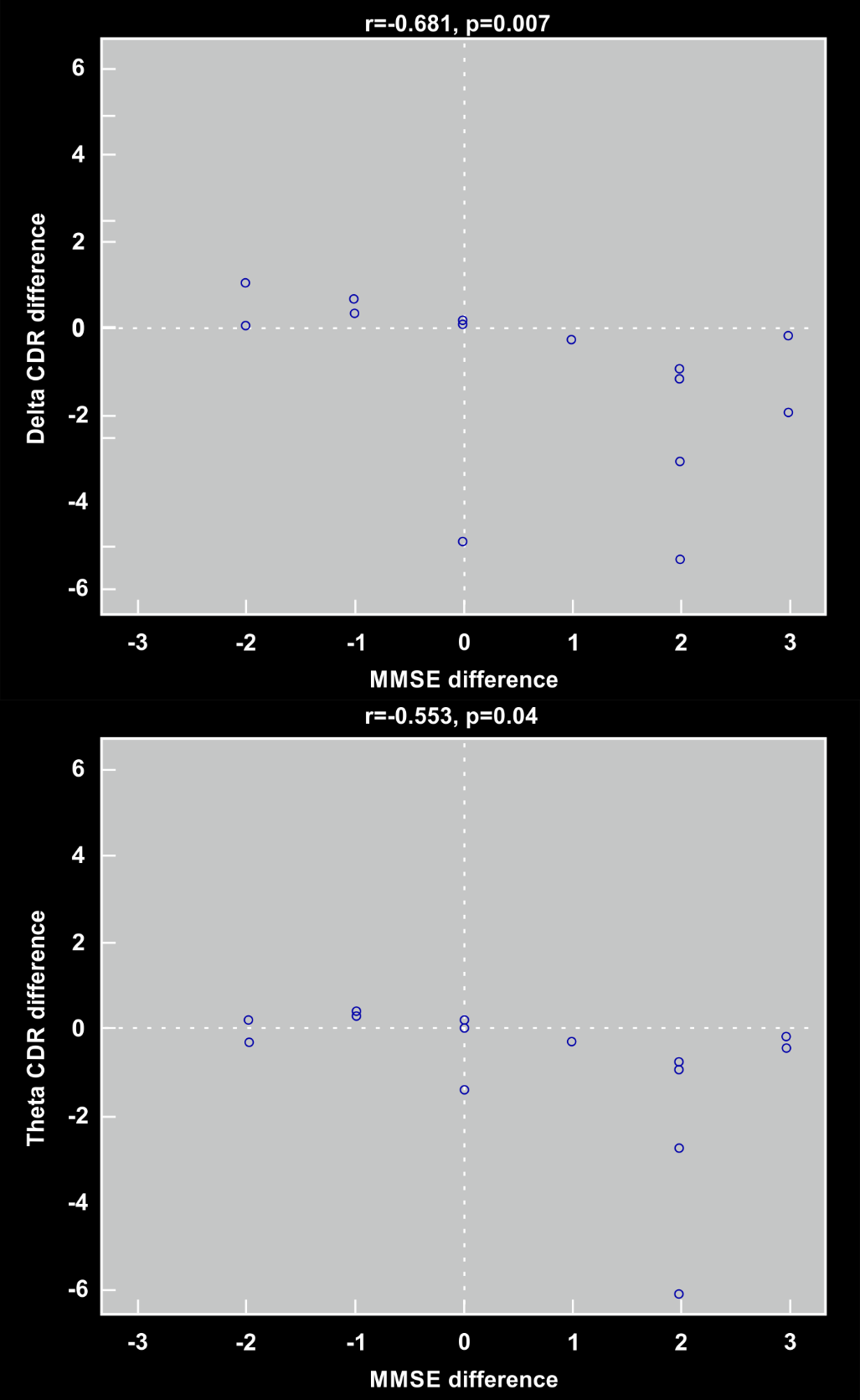
**

**Supplementary Figure Captions**

**Supplementary Figure 1.** Grand average of eLORETA solutions (i.e. CDR at PCu/PCC voxels at p<0.05, corrected) modelling the EEG source for theta band in the new sample of the LLM group on the corresponding axial (left view) and sagittal (right view) generic MRI slices. The left side of the maps (left view) corresponds to the left hemisphere. The power estimate was scaled based on the averaged maximum value indicated in the scale bar. Similar illustrations but of fewer voxels apply for the delta, and beta1 bands.

**Supplementary Figure 2.** Visualization of the negative correlation of delta (r = -0.681, p = 0.007) (top view) and theta bands (r = -0.553, p = 0.04) (bottom view) among the MMSE score post to pre difference of each participant and the CDR post to pre difference of PCu/PCC activity that was statistically significant at p<0.05, corrected.

**Supplementary Tables**

**Supplementary Table 1.** Subject Pool (Means ± SDs) for new LLM sample

|  | **LLM** |
| --- | --- |
| **no. of subjects** | 14 |
| **no. of males/ratio** | 4 (28.57%) |
| **age** | 66.92 ±6.06 |
| **MMSE pre** | 25.71 ±1.03 |
| **MMSE post** | 26.28 ±1.22 |
| **yoe** | 7.42±2.66 |
| **duration** | PT:25.85±7.33 h  CT:29.71±7.13 h |

**Supplementary Table 2.** EEG source maps at the differences at *p<* 0.05, corrected

| **LLM replication sample** | | | | | |
| --- | --- | --- | --- | --- | --- |
| **Band** | **Anatomical area** | **BA** | **CS** | **F** | **MNI coordinates (mm)**  **x,y,z** |
| **Delta (2-4 Hz)** | PCu/PCC | 31 | 4 | 2.9842 | 25,-75,20 |
| **Theta (4-8 Hz)** | PCu/PCC | 31 | 4 | 3.1279 | 25,-75,20 |
| **Beta1 (12-18 Hz)** | PCu/PCC | 31 | 2 | 2.7869 | 20,-75,20 |
| **Beta2 (18-30 Hz)** | Superior Temporal Gyrus | 22 | 46 | 2.6911 | 45 -10 -5 |
|  | PCu/PCC | 31 | - | 2.2322* | 20,-75,20 |

Note: Results are superimposed on standardized MNI coordinates; BA, Brodmann Area; x, left/right; y, anterior/posterior; z, superior/inferior; CS, cluster size in number of activated voxels; F, F-value; T, t-value; PCu/PCC, precuneus/posterior cingulate cortex; Signiﬁcant at *p<*0.05 corrected; *; no significance.
